# Highly efficient multiplex genetic engineering of porcine primary fetal fibroblasts

**DOI:** 10.1016/j.sopen.2020.11.003

**Published:** 2020-11-18

**Authors:** Benjamin Klapholz, Heather Levy, Ramesh Kumbha, Nora Hosny, Michael E. D'Angelo, Bernhard J. Hering, Christopher Burlak

**Affiliations:** a Horizon Discovery, 8100 Cambridge Research Park, Waterbeach, Cambridge CB25 9TL, UK; b Schulze Diabetes Institute, Department of Surgery, University of Minnesota Medical School, Minneapolis, MN, USA; c Department of Surgery, Schulze Diabetes Institute, University of Minnesota School of Medicine, Minneapolis, MN, USA; d Department of Medical Biochemistry and Molecular Biology, Faculty of Medicine, Suez Canal University, Ismailia, Egypt

## Abstract

**Background:**

Genetically engineered porcine donors are a potential solution for the shortage of human organs for transplantation. Incompatibilities between humans and porcine donors are largely due to carbohydrate xenoantigens on the surface of porcine cells, provoking an immune response which leads to xenograft rejection.

**Materials and Methods:**

Multiplex genetic knockout of GGTA1, β4GalNT2, and CMAH is predicted to increase the rate of xenograft survival, as described previously for GGTA1. In this study, the clustered regularly interspaced short palindromic repeats/clustered regularly interspaced short palindromic repeats–associated protein 9 system was used to target genes relevant to xenotransplantation, and a method for highly efficient editing of multiple genes in primary porcine fibroblasts was described.

**Results:**

Editing efficiencies greater than 85% were achieved for knockout of GGTA1, β4GalNT2, and CMAH.

**Conclusion:**

The high-efficiency protocol presented here reduces scale and cost while accelerating the production of genetically engineered primary porcine fibroblast cells for in vitro studies and the production of animal models.

## INTRODUCTION

Genetic engineering offers the possibility of transforming the treatment of several diseases. A particularly significant application of genetic engineering is xenotransplantation (transplantation between species). Porcine organs are preferred for transplant to humans, and their use in the clinical setting could provide a potential solution to end the donor organ shortage [[1], [2], [3]]. The recipient's immune system prevents xenograft survival due to the expression of epitopes found on the surfaces of porcine cells, evoking hyperacute rejection via activation of the complement cascade [[3], [4], [5]]. Modifications to porcine xenografts are intended to bypass the human immune system for the purpose of cloning porcine donors without the genes encoding for the antigenic glycans [4,6]. Following a double-stranded break (DSB) in DNA, repair made by nonhomologous end joining (NHEJ) can lead to base pair (bp) insertions and deletions (indels), resulting in successful gene knockout (KO) [7]. Improper translation due to these indels subsequently impairs the production of associated proteins. Multiplexed KO of particular genes (*GGTA1*, *β4GalNT2*, and *CMAH*) in porcine cells could allow for longer graft survival [8]. This paper introduces an optimized protocol for multiplex KO in xenotransplant using the clustered regularly interspaced short palindromic repeats (CRISPR)/CRISPR-associated protein 9 (Cas9) gene editing system.

Gene editing by NHEJ was a slow process by today's standards, and through breeding and selectable markers, it was shown to be possible to produce animals for research [[9], [10], [11]]. The emergence of zinc finger nucleases (ZFNs) and transcription activator-like effector nucleases (TALENs) was a critical step in the development of targeted gene editing. Both ZFNs and TALENs are programmable and can be custom designed via attachment of a restriction endonuclease to cut DNA at a desired location. In the case of TALENs, each endonuclease recognizes 1 nucleotide at a time, making them cheaper and easier to assemble than ZFNs; in addition, TALENs have been shown to produce less off-target effects [12]. Despite these advancements, both ZFNs and TALENs are expensive and laborious methods for targeting specific sequences for gene KO.

The recent discovery of the CRISPR gene editing system [13,14] and the subsequent use of CRISPR associated (Cas) endonucleases have irreversibly changed the field of genetic engineering. To achieve targeted genetic modification, Cas9 makes a DSB 3 nucleotides upstream of a protospacer adjacent motif (PAM); in the case of Cas9, the PAM site is NGG. With the addition of multiple single-guide RNAs (sgRNAs), the CRISPR/Cas9 system has the impressive ability to edit multiple genes at once [9]. Given the rapid acceptance of CRISPR/Cas9, multiple vendors are competing to provide the best transfection material for gene KO research. Numerous groups have sought to develop an optimal CRISPR/Cas9 protocol to maximize gene editing efficiency and minimize off-target effects, leading to various modifications of current systems [[15], [16], [17]]. A sample of past KO research in the field of xenotransplantation, including multiple transfection systems and the transfection efficiency achieved, is shown in Table 1. Values displayed in Table 1 demonstrate efficiencies prior to selection methods such as antibiotic or affinity column selection. For the majority of the articles cited in Table 1, sequencing and tracking of indels by decomposition (TIDE) analysis were not available.

**Table 1:** Historic transfection efficiencies of GGTA1 based on phenotypical analysis

*Gene editing system*	*Transfection system*	*Phenotype transfection efficiency*	*Authors (year)*
ZFNs	Gene Pulser Xcell	1%	Hauschild et al (2011) [29]
TALENs	BTX Legacy ECM 2001	5.0%	Yao et al (2014) [30]
CRISPR/Cas9	Amaxa 4D-Nucleofector	1.7%	Sato et al (2015) [31]
CRISPR/Cas9	Neon	55.2%	Li et al (2015) [9]
TALENs	Gene Pulser Xcell™	7.1%	Cheng et al (2016) [32]
TALENs	Amaxa 4D-Nucleofector	53.7%	Kang et al (2016) [33]

In the present study, a superior protocol is proposed for genetically engineering porcine fetal fibroblasts for subsequent use in in vitro analysis or the cloning of genetically engineered KO porcine donors. Other factors, including confluency, incubation conditions, and cell media, are also provided. Single and multiplexed KOs were analyzed by flow cytometry, and Sanger sequencing traces were studied by TIDE analysis (https://tide.deskgen.com). Results of this study demonstrate the improved gene editing efficiency provided by the proposed standard operating procedure (SOP) (Supplementary Material).

## METHODS AND MATERIALS

### sgRNA Design

Algorithms for acceptable target sites were found; sgRNAs were designed using the integrated Benchling CRISPR gRNA Design tool (https://benchling.com/crispr) for *β4GalNT2* and the ZiFiT Targeter tool (http://zifit.partners.org/ZiFiT/) [19,20] for *GGTA1* and *CMAH*. The sgRNA for *β4GalNT2* was designed to cut both paralogues.

The following sequences were targeted (5′–3′):

GGTA1: GCTGCTTGTCTCAACTGTAA

CMAH: ATGAAGTATATCAATCCTCC

β4GalNT2 E2: ACATAAAGAGTCCAACGCTC

β4GalNT2 E3: GATGCCCGAAGGCGTCACAT

β4GalNT2 E9: CGTCCTAGAGAAAACGGAAC

### Transfection of Primary Porcine Fetal Fibroblasts

A combination of modified synthetic sgRNA, high-grade Cas9 protein (sNLS-SpCas9-sNLS), and a Nucleofector Transfection System was used to optimize CRISPR/Cas9 gene editing efficiency. Primary porcine fetal fibroblast cells obtained from Mangalista cell line 41 were used for transfection. Media were removed and cells were washed with Dulbecco's phosphate-buffered saline (DPBS) (#9212, Gibco). TrypLE Express (#12604-021, Gibco) was used to harvest cells at 37°C for 5 to 8 minutes. Complete Nucleofector SE solution was prepared according to manufacturer instructions included with the Amaxa SE Cell Line Optimization 4D-Nucleofector X Kit (#V4XC-9064, Lonza) according to manufacturer instructions (Lonza). Briefly, Nucleofector SE solution was prepared by combining 82 μL of SE 4D-Nucleofector X Solution and 18 μL of supplement solution for a total volume of 100 μL per transfection. A 1.25-μL aliquot of Aldevron Cas9 (sNLS-SpCas9-sNLS) (10 μg/μL) was combined with 3.1 μL of Synthego sgRNA (150 μmol/L) in a 200-μL microfuge tube. At room temperature, 5 × 10^4^ cells were pipetted gently into the prepared Complete Nucleofector SE solution using a P1000 tip. Using a P200 tip, a total volume of 100 μL was transferred to a ribonucleoprotein (RNP)–containing tube prior to being mixed one time, gently. A total volume of 100 μL was then pipetted into a nucleofection cuvette so that no bubbles appeared. The bottom of the nucleofection cuvette was tapped, and the cuvette was placed into the Amaxa 4D-Nucleofector Transfection System unit. Program CM-137 was used for transfection. Cells were not left in SE solution for longer than 10 minutes. Following transfection, a volume of 500 μL of prewarmed Dulbecco's modified eagle medium (DMEM) with 20% fetal bovine serum (FBS) cell culture media was added to each nucleofected sample without mixing. Samples were then incubated at 37°C for 10 to 15 minutes. Using long-nosed plastic pipettes, transfected cells were very gently transferred to 6-well plates containing prewarmed 20% FBS cell culture media. Cells were then incubated at 37°C for 18 to 24 hours before media were replaced with DMEM + 10% FBS media. For further details, refer to the SOP (Supplementary Material).

### Phenotyping

Phenotypical analysis and sorting of cells were performed using a BD FACSVerse Flow Cytometer (651153, BDBioSciences) according to manufacturer instructions (BDBioSciences). Transfected and wild-type cells were harvested, washed, and then incubated individually or in combinations of isolectin B_4_ (IB_4_), N-glycolylneuraminic acid (Neu5Gc), and *Dolichos biflorus* agglutinin (DBA). IB_4_ was at a concentration of 0.5 μL in 100 μL DPBS + 5% FCS, and cells were stained for 30 minutes on ice. Neu5Gc was made using 0.5% Neu5GC Assay Blocking Solution in DPBS at 500 μL per sample, and DBA was prepared in 1 μL per 100 μL DMEM + 10 mmol/L calcium chloride and stained for at least an hour on ice. The following biotinylated isolectin conjugates were used for IB_4_ staining: Isolectin GS-IB_4_ from *Griffonia simplicifolia,* Alexa Fluor 488 Conjugate (#I21411, Thermo-Fisher), 647 Conjugate (#I32450, Thermo-Fisher), and 568 Conjugate (I21412, Thermo-Fisher). The following labeled lectin conjugates were used for DBA staining: fluorescein-labeled DBA (#FL-1031, Vector Labs) and rhodamine-labeled DBA (#RL-1032, Vector Labs).

### Genotyping

Each gene of interest was amplified by polymerase chain reaction and isolated for sequencing, and the following primers were used (5′–3′):

GGTA1:

Forward: CCTTAGCGCTCGTTGACTATTC

Reverse: TTTCTTTGCTTTTTAGGGCCGC

CMAH:

Forward: ATGGCTCTGCTGATCTCTAACA

Reverse: TCATCTCATTTACGCCGACTCT

β4GalNT2 E2:

Forward: TGTGATCAGAAGTGCGTATTTGAA

Reverse: AAGGACACAGTAAAGCCACAG

β4GalNT2 E3:

Forward: CTGGGATTCCAGGGTCTCAAC

Reverse: ACACCCTCGGGAATGAGTAGA

β4GalNT2 E9:

Forward: TTCCCGGAGAAATCAGGTCAC

Reverse: CCTCCCCCTCTGGCTCG

The following primers were used during TIDE analysis (5′–3′):

GGTA1: TTTCTTTGCTTTTTAGGGCCGC

CMAH: ATGGCTCTGCTGATCTCTAACA

β4GalNT2 E2: AAGGACACAGTAAAGCCACAG

β4GalNT2 E3: ACACCCTCGGGAATGAGTAGA

β4GalNT2 E9: CCTCCCCCTCTGGCTCG

## RESULTS AND DISCUSSION

### Guide Optimization

**Fig 1:**
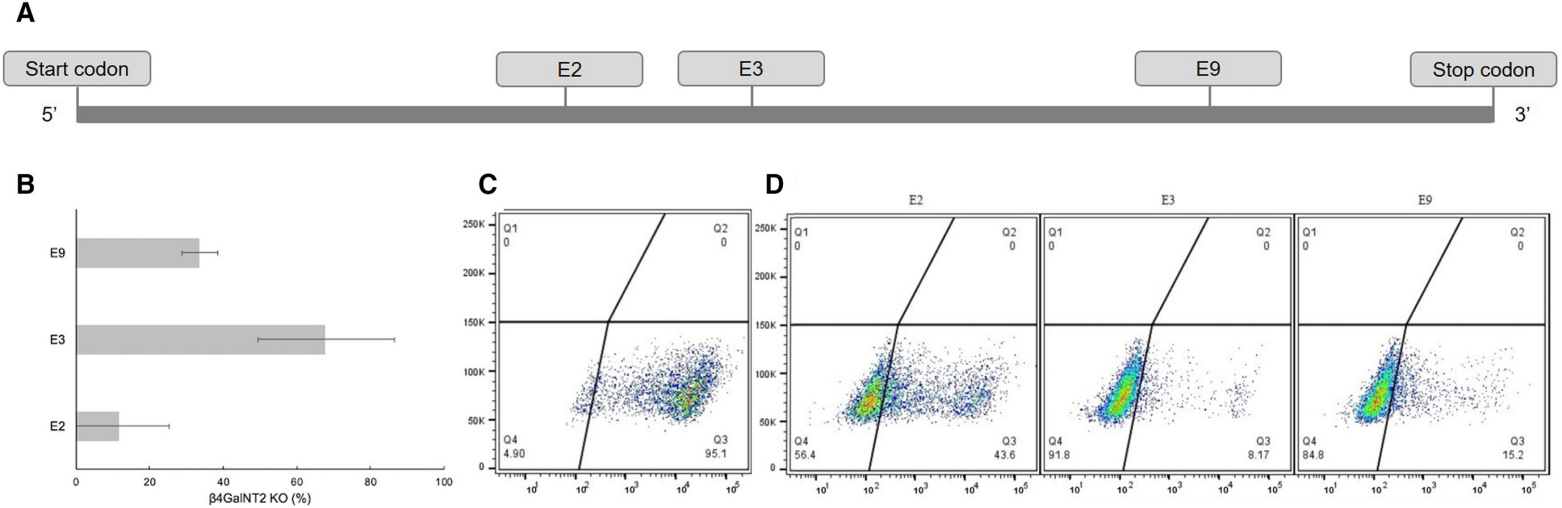
Optimization of β4GalNT2. Three sgRNA were designed and tested to determine an optimal cut site for β4GalNT2 transfections. (A) A gene map demonstrates candidate sgRNA templates for cut sites in the β4GalNT2 gene. (B) Gene editing efficiency for various β4GalNT2 cut sites is shown by TIDE analysis. A region in E3 and E2 was each tested 3 times, whereas 1 region in E9 was tested twice. Average efficiency is shown with standard deviation displayed as error. (C-D) Phenotype data for cells stained with DBA-lectin are shown with flow cytometry–generated density plots. (C) A porcine WT control is shown. (D) Phenotype data for cells transfected with E2, E3, and E9 are shown.

In the context of the CRISPR/Cas9 gene editing system, a critical factor for editing cells is to attain a very high transfection efficiency without compromising cell viability. An optimization procedure can be used to demonstrate which sgRNAs will lead to the highest proportion of genetically engineered cells for a given cut site. For example, potential cut sites in the coding sequence of *β4GalNT2*, within exons 2 (E2), 3 (E3), and 9 (E9), are shown (Fig 1, *A*) [18]. Insertions and deletions at the targeted sites on DNA can be observed by TIDE analysis, which will indicate gene editing efficiency (%) for each sgRNA tested. Efficient gene KO will be correlated to a high editing frequency. Results of TIDE analyses are also shown (Fig 1, *B*). Gene editing of E2 and E3 was tested 3 times each, and gene editing of E9 was tested twice. E2 showed the lowest editing frequency compared to E3 and E9 (Fig 1, *B*). TIDE analysis of E9 indicated that only one third of cells had altered sequences in the region of interest and E3 had an average gene editing frequency of 67.9% (Fig 1, *B*), although a frequency of 89.2% was achieved in 1 trial.

Phenotype reflects function of a gene; efficient gene editing aims to eliminate expression of the gene of interest. As seen in Fig 1, *C* and *D*, cells are probed with a DBA-lectin stain which specifically labels cells expressing β4GalNT2. Ninety-five percent of primary porcine embryonic fibroblasts naturally express β4GalNT2 (Fig 1, *C*). In Fig 1, *D*, flow cytometry phenotype density plots are shown for 1 trial of each of the 3 β4GalNT2 cut sites. Cells transfected with an E2-targeting Cas9-RNP show a fairly even distribution between negative and positive expression of β4GalNT2 (Fig 1, *D*). In comparison, a larger proportion of cells transfected with the E3- or E9-targeting Cas9-RNP had low β4GalNT2 levels (Fig 1, *D*). E9-targeting Cas9-RNP resulted in a much higher phenotype KO than suggested by the TIDE analysis (Fig 1, *D*). The E3 cut site was shown to have the highest deletion frequency and an adequate standard deviation, as well as the highest phenotype KO efficiency (Fig 1, *B*, *D*). Therefore, sgRNA utilized for targeted modification of E3 was determined to be the optimal sgRNA for β4GalNT2 KO.

### Novel Single KO Efficiency

**Fig 2:**
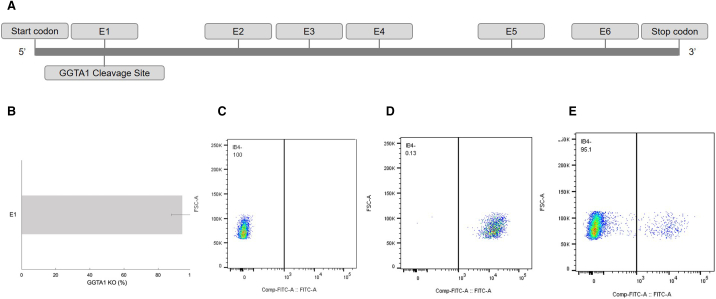
Highly efficient GGTA1 KO. Porcine cells were transfected with preoptimized sgRNA targeting the GGTA1 gene and compared to WT cells. (A) A gene map demonstrates the specific cut site in E1 for GGTA1. (B) Gene editing frequency at this cut site is shown by TIDE analysis. Two replicates were performed. Most of the cells have a deletion of 1 bp, and in 1 trial, an editing frequency of 98.7% was achieved. (C-E) Phenotype data are shown by flow cytometry–generated density plots. Cells were either unstained or stained with IB4-lectin. (C) An unstained porcine WT control is shown. There are not any false positives. (D) A porcine WT control is shown. A small population of WT cells naturally does not express GGTA1. (E) Most transfected cells were not labeled by the IB4-lectin, demonstrating that most did not express GGTA1.

Data shown in Fig 2 demonstrate remarkably high gene editing efficiency for engineering *GGTA1* KO porcine fibroblast cells with the provided SOP. Following cut site optimization, as described for *β4GalNT2* (Fig 1), porcine cells were transfected with a Cas9-RNP targeting exon 1 (E1) of *GGTA1*. Potential cut sites for GGTA1 KO, including E1, are shown (Fig 2, *A*). Cells were probed with an IB_4_-lectin that only labels cells expressing *GGTA1*. An unstained porcine WT control used to define false positives and negative gates is also shown (Fig 2, *C*). To verify KO, expression levels of GGTA1 were quantified, comparing KO cells (Fig 2, *E*) to normal porcine WT expression (Fig 2, *D*). Figure 2, *D* demonstrates that very few WT cells (0.13%) were naturally negative for IB_4_-lectin, whereas Fig 2, *E* presents evidence that 95.1% of the edited cells lost *GGTA1* activity. The TIDE analysis shown in Fig 2, *B* also reflects these data. When sequenced and compared to the same region in WT cells, most of the cells contained an insertion or a deletion of 1 or more bp, effectively resulting in gene KO. In this study, TIDE analysis showed a gene editing efficiency of 98.7% (Fig 2, *B*) and flow cytometry showed a phenotype KO of 95% (Fig 2, *C*).

### Novel Multiplex KO Efficiency

**Fig 3:**
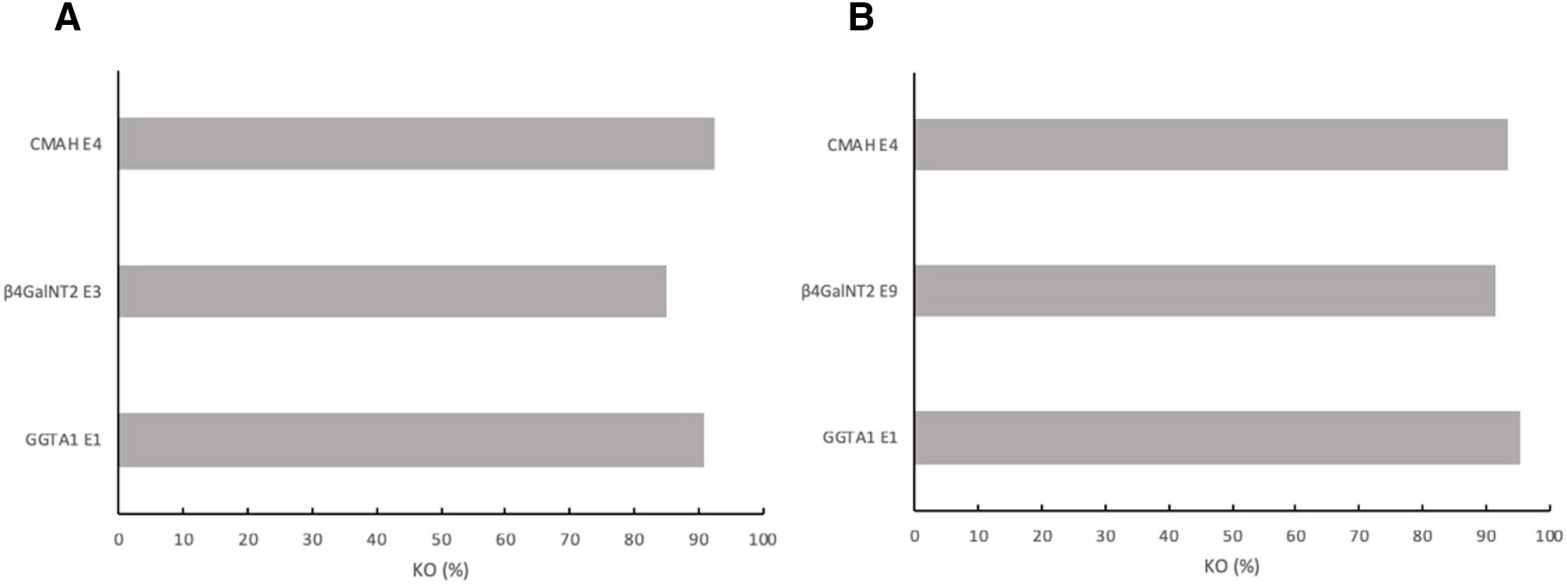
Highly efficient GGTA1, CMAH, and β4GalNT2 multiplex KO. Analysis of gene editing efficiencies determined by TIDE data on modified cells is shown. (A) Multiplexed β4GalNT2 (E3), CMAH, and GGTA1 gene editing. (B) Multiplexed β4GalNT2 (E9), CMAH, and GGTA1 gene editing.

Very high gene editing efficiencies were observed when using the optimized protocol for multiplexed Cas9-RNPs, targeting multiple genes. Data shown in Fig 3 demonstrate results of 2 transfections conducted during this study. Transfection was performed with CMAH, β4GalNT2 (E3), and GGTA1 (Fig 3, *A*), and TIDE analysis demonstrated efficiencies of 92.5%, 84.9%, and 91%, respectively, for the modified cells (Fig 3, *A*). Similar transfection was performed with β4GalNT2 (E9) replacing β4GalNT2 (E3) (Fig 3, *B*). In this study, editing efficiencies were found to be slightly elevated; GGTA1 had an efficiency of 95.4%, β4GalNT2 (E9) had 91.5%, and CMAH had an efficiency of 93.6% (Fig 3, *B*). Results of both transfections performed in this study show an excitingly high ability for the optimized protocol to produce genetically engineered cells.

The use of sgRNA in this experiment, as opposed to chimeric pX330 guide RNA expression plasmids, is a key feature of the supplied SOP for transfecting porcine fibroblast cells with high efficiency [22]. Production of *GGTA1* KO cells was previously achieved with an efficiency of 55.2% by transfection of liver-derived porcine cells with pX330 plasmids and CRISPR/Cas9 [9]. Similarly, a proposed CRISPR/Cas9 protocol for gene editing demonstrated a high average editing efficiency of 60% across multiple genes [21]. The results of the present study demonstrate reproducible editing efficiencies well above 90% (Fig 1, Fig 2, Fig 3).

Numerous protocols are available to researchers for the production of genetically engineered KO cells when purchasing the materials needed for transfection. Synthego produces customized experimental protocols for researchers and guarantees at least 50% KO efficiency in human cell lines. No guarantee is made for other types of cells, that is, mammalian and stem cells; however, this provides a significant opportunity for the scientific community to experiment, optimize, and share CRISPR protocols. The Aldevron Cas9 used in this study was selected due to its WT SpCas9 region and Good Manufacturing Practices classification. The sgRNAs utilized in these experiments were purchased from Synthego because chemical modifications to analogs were allowed during the purchase, allowing for the RNP to last longer in the cell by increasing durability and adding protection against the intracellular immune system. Moreover, whereas some manufacturers include modifications as well, Synthego reports purer sgRNA.

Multiple concerns exist regarding the use of CRISPR/Cas9 in gene editing. Published KO efficiencies are highly variable depending on the protocol used, given the abundance of factors that contribute to efficient editing, including cell line, proximity of a PAM sequence, cell confluency, electroporation or nucleofection efficiency, type of Cas9, incubation conditions, etc. Although the proposed protocol provides solutions for several of these issues, other challenges remain unresolved. The threat presented by the occurrence of off-target effects, as seen in homologous recombination, ZFN, and TALENs systems, is significant enough to slow research using CRISPR/Cas9; however, current efforts are focused on reducing the likelihood of unintended genetic mutations. Whether it is possible to find an optimized protocol that works at high efficiency across all possible types of cells and genes is debatable, and CRISPR/Cas9 protocols may have to be tailored to particular genes of interest [23]. Despite such limitations, the optimized protocol presented here is applicable for studying and engineering models for xenotransplantation, with additional applications in animal models, stem cells, and gene therapy, as well as others [[24], [25], [26], [27], [28]]. Increased gene editing efficiencies could substantially decrease the amount of time needed for cell sorting, providing more time for novel research and discovery.

In conclusion, the production of genetically modified cells is challenging and time consuming; however, with the growing use of the CRISPR/Cas gene editing system and optimization of related protocols, multiplex KO cells can be prepared with high efficiency in a matter of days. The SOP included in this study is intended to aid in improving gene editing efficiencies for a wide range of applications, allowing researchers to focus on identifying genes of interest and improving cloning processes rather than being limited by historically used techniques with low gene editing efficiencies.

## Author Contribution

BK performed critical experiments, analyzed data, and performed a critical review of manuscript; HL, RK, and MD performed experiments; BH performed critical review of manuscript; and CB designed experiments, performed critical analysis, and wrote manuscript.

## Conflict of Interest

B.J.H. has an equity interest in and serves as an executive officer of Diabetes-Free, an organization that may commercially benefit from the results of this research. This interest has been reviewed and managed by the University of Minnesota in accordance with its Conflict of Interest policies. The other authors declare no competing interests.

## Funding Source

Genetic editing of porcine primary embryonic fibroblasts was supported by a sponsored research agreement between Diabetes-Free, Inc, and the 10.13039/100007249University of Minnesota.

We would like to thank Anna Lucia Krupp, MS, for assistance with writing and editing this manuscript.

## Appendix A Supplementary data

Supplementary material 1 Image 1

Supplementary material 2 Image 2
