# Supplementary material for: Highly efficient multiplex genetic engineering of porcine primary fetal fibroblasts
Source: Surg Open Sci. 2020 Nov 18;4:26–31. doi: 10.1016/j.sopen.2020.11.003 (PMC8074785; doi:10.1016/j.sopen.2020.11.003)
Supplement: Supplementary file 1 — Supplementary material 1 [file mmc1.docx]

**1. PURPOSE**

1.1 To describe the procedure steps for the transfection of porcine fetal fibroblast cells using Aldevron Cas9, Synthego synthetic single guide RNA (sgRNA), and the Lonza 4D-NucleofectorTM Transfection system to generate knockout (KO) cells.

**2. DEFINITIONS**

2.1 DEHS Department of Environmental Health and Safety

2.2 DMEM Dulbecco’s modified eagle medium

2.2 DPBS Dulbecco’s phosphate buffered saline

2.3 KO knockout

2.4 PPE personal protective equipment

2.5 SDI Schulze Diabetes Institute

2.6 sgRNA single guide RNA

2.7 SOP standard operating procedure

**3. EQUIPMENT**

3.1 Drummond Pipet-aid

3.2 Cell Incubator Centrifuge

3.3 Water Bath

3.4 Microscope

3.5 Eppendorf Pipettes, assorted

3.6 Lonza 4D-Nucleofector^TM^ System (Amaxa)

**4. MATERIALS**

4.1 sNLS-SpCas9-sNLS Cas9 Nuclease; stock at 10µg/µl (Aldevron, #9219)

4.2 Costar Cell Culture Plate, 6 wells (Corning, #3506)

4.3 Costar Cell Culture Plate, 24 wells (Corning, #3527)

4.4 75cm² Cell Culture Flask, canted neck (Corning, #431464U)

4.5 Costar Microcentrifuge Tubes (Corning, #3213)

4.6 Falcon 15mL Polypropylene Conical Tube (#352097)

4.7 Gibco DPBS (Thermo-Fisher Scientific, #14040-133)

4.8 Gibco Fetal Bovine Performance Plus (Thermo-Fisher Scientific, #10082147)

4.9 Gibco GlutaMAX (Thermo-Fisher Scientific, #35050-061)

4.10 Gibco TrypLE Express (Thermo-Fisher Scientific, #12604-021)

4.11 Lonza SE Cell Line Optimization 4D-Nucleofector X Kit (Amaxa, #V4XC-1024)

4.12 sgRNA (Synthego)

4.13 DMEM (Thermo-Fisher Scientific, #31053028)

4.14 Penicillin-Streptomycin (Thermo-Fisher Scientific, #15140122)

**5. PROCEDURE**

5.1 Prepare the following:

10% FBS Media

88% DMEM

10% FBS

1% P-S

1% GlutaMAX

20% FBS Media

78% DMEM

20% FBS

1% P-S

1% GlutaMAX

5.2 Prepare wells of 6-well plate(s) with 1.5ml 20% FBS media and place at 37˚C

5.3 Place 20% FBS media into 37°C water bath

5.4 Start 4D-Nucleofector^TM^ system and enter program CM-137

5.5 Prepare Complete Nucleofector SE per transfection and keep at room temperature:

82 µL SE solution

18 µL supplement

For each sgRNA needed:

5.6 Prepare ribonucleoprotein (RNP) complexes separately in 200 µL tubes ^I^:

1.25 µL Cas9 protein (10 µg/µL)

3.1 µL sgRNA (150 µM)

*^I^ Volume is too small to mix by vortex, mix by stirring with pipette tip. Check tip for residual volume before ejecting.*

5.7 Incubate for 10 min. at room temperature and then keep on ice until use ^II^

*^II^ Do not mix different RNPs at this point!*

5.8 Remove media from cells in flasks

5.9 Wash cells in 10 mL DPBS

5.10 Add 1.5ml TrypLE Express to cells, then incubate for 5 to 8 min. at 37°C ^III^

*^III^ Cells can’t stay too long in TrypLE Express - 8 min. max. Some cells may not detach.*

5.11 Add 8.5ml of 10% FBS media to each flask

5.12 Resuspend cells by pipetting up and down before transferring to 15ml tube

5.13 Count cells with hemacytometer

5.14 For nucleofection in cuvettes, calculate required volume for 500,000 cells/nucleofection

5.15 Transfer required volume to 15ml tube

5.16 Spin down cells at 200 xg for 4 min. at room temperature before discarding supernatant

5.17 Resuspend cells in 10 mL room temperature DPBS

5.18 Spin down cells at 200 xg for 4 min. at room temperature

For multiplexed KO:

5.19 While spinning cells with DPBS, pool RNPs in 200 µl tubes

5.20 Discard most of DPBS supernatant with 10 mL pipette

5.21 Use P1000 tip to remove as much DPBS as possible

5.22 Place 200µl tubes containing RNPs under the hood at room temperature

5.23 Resuspend cells in Complete Nucleofector SE solution by gently pipetting up and down with P1000 micropipette

5.24 Transfer cells in Complete Nucleofector SE solution to sterile 1.5 mL tube ^IV^

^IV^ *Cells can’t stay too long in SE solution – 10 min. max.*

For each nucleofection:

5.25 Use P200 micropipette to add 100 µl cells/SE solution to each RNP-containing 200 µL tube

5.26 Mix cells + RNPs by pipetting up and down gently ONCE only

5.27 Transfer 100 µL cells/SE solution/RNPs to cuvette, making sure to leave NO bubbles

5.28 Tap cuvette gently to make sure liquid covers bottom surface

5.29 Place cuvette in Lonza Nucleofector^TM^ X-unit

5.30 Start Program CM-137 to initiate nucleofection

5.31 Add 500 µL warm 20% FBS media to each nucleofection cuvette - *do not mix!*

5.32 Incubate nucleofected cells in cuvettes at 37°C for 10 to 15 min.

5.33 Using provided plastic pipette, gently transfer cells to prewarmed 6- or 24-well plate(s)

5.34 Incubate plate(s) at 37°C for 18 to 24 h.

5.35 Change media in each well, replacing 20% FBS media with 10% FBS media
