# Supplementary figures and images for: Highly efficient multiplex genetic engineering of porcine primary fetal fibroblasts
Source: Surg Open Sci. 2020 Nov 18;4:26–31. doi: 10.1016/j.sopen.2020.11.003 (PMC8074785; doi:10.1016/j.sopen.2020.11.003)

## Slide 1
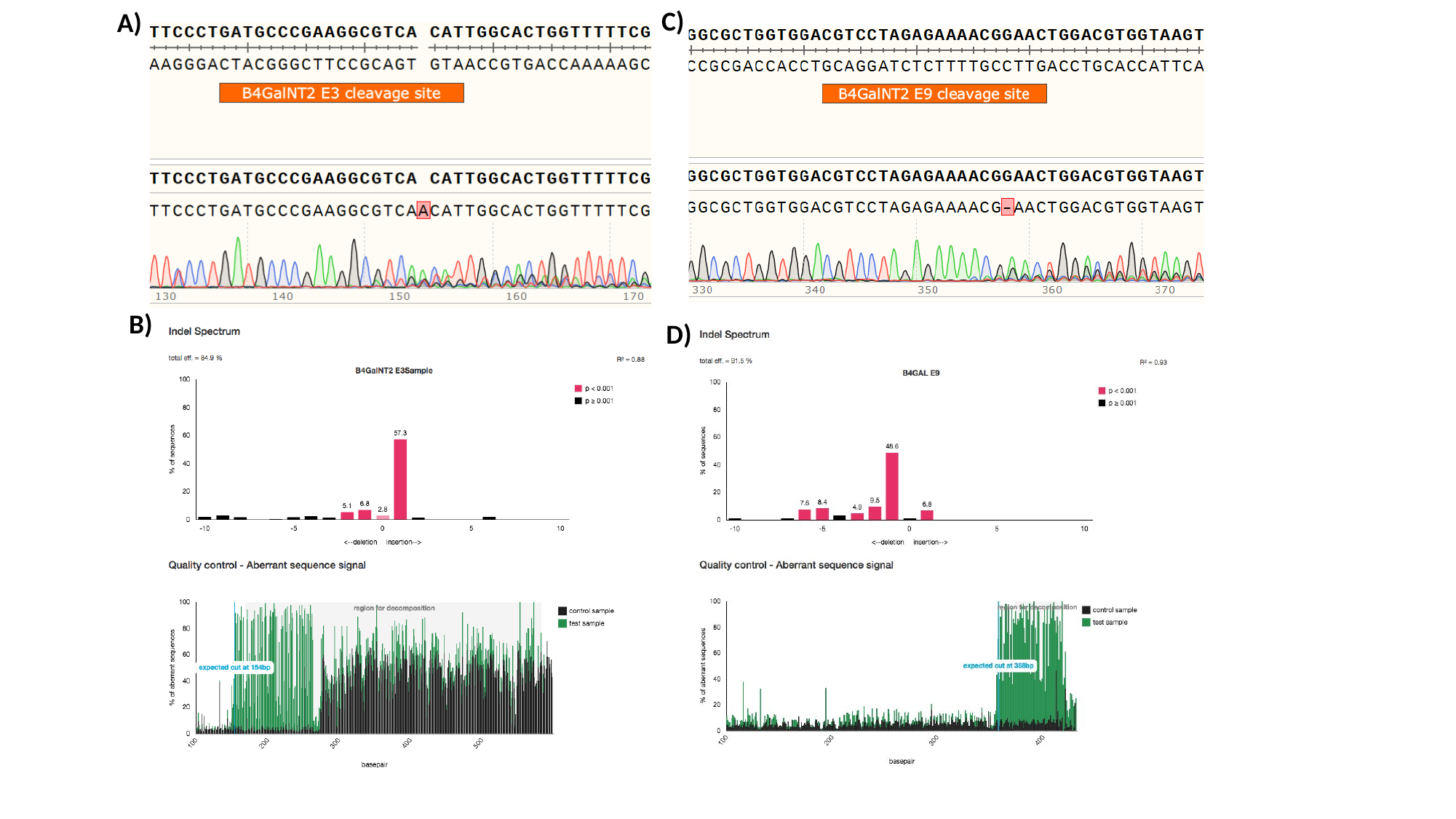

A)
B)
C)
D)

## Slide 2
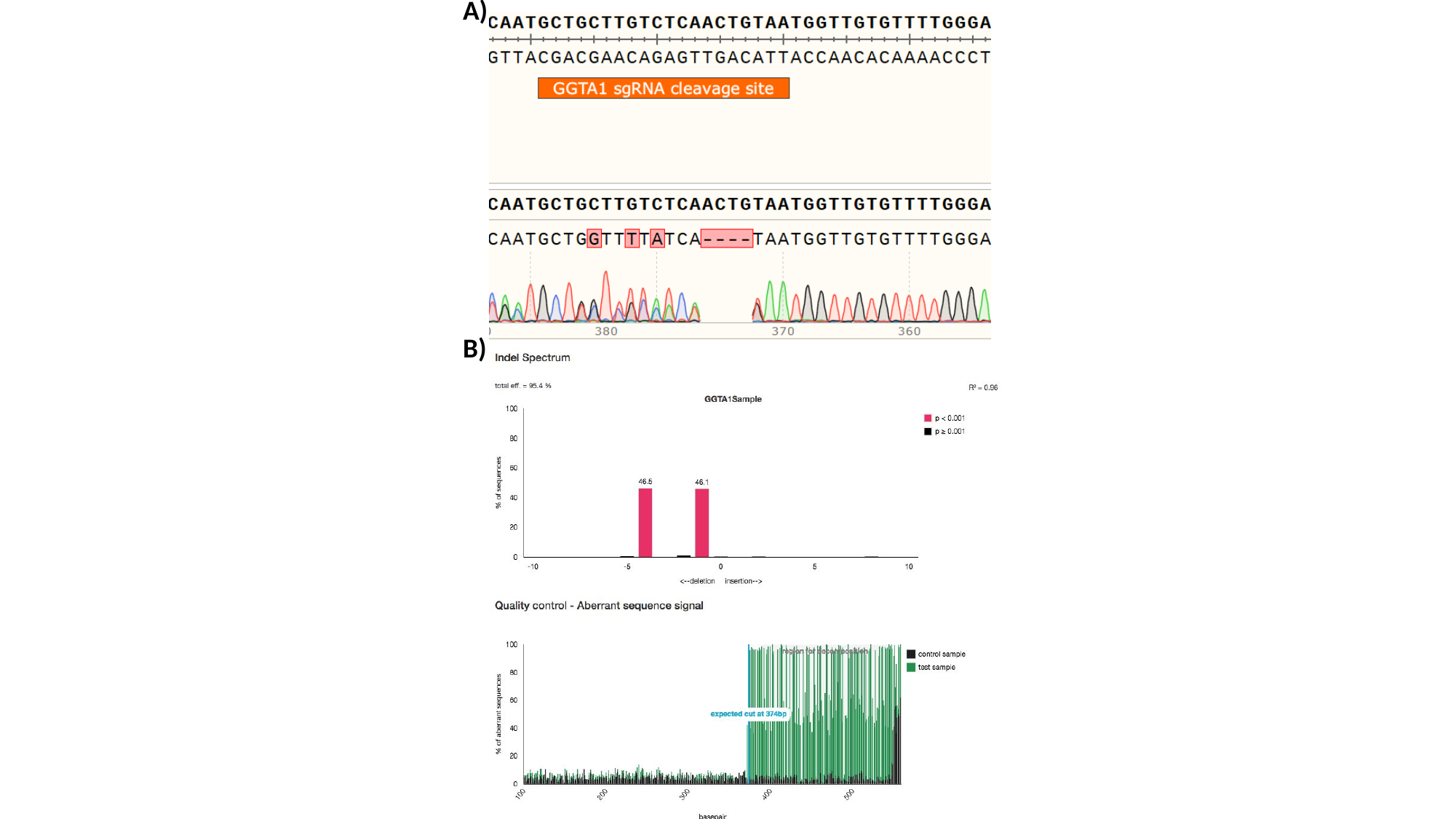

A)
B)

## Slide 3
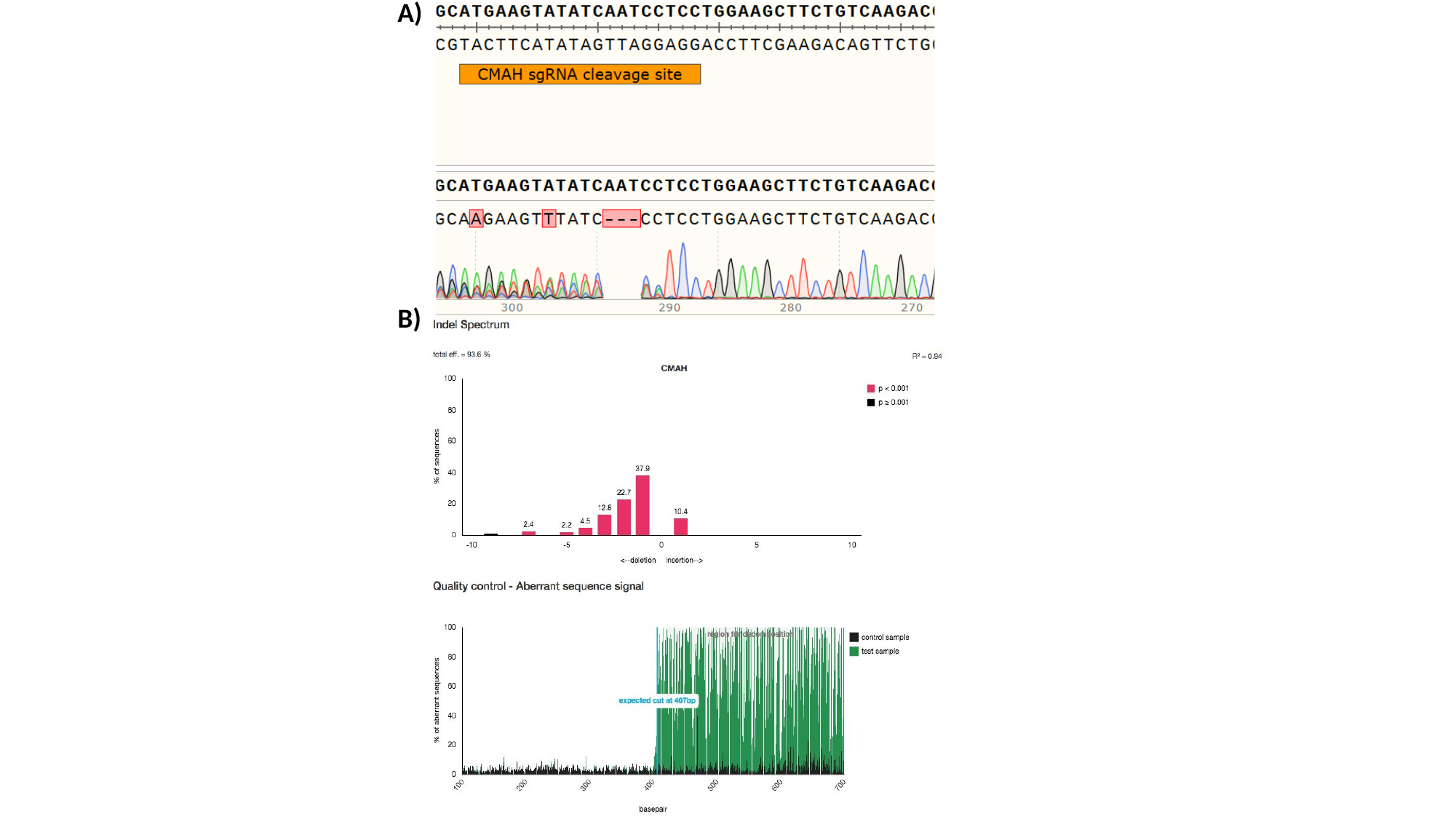

A)
B)

## Slide 4
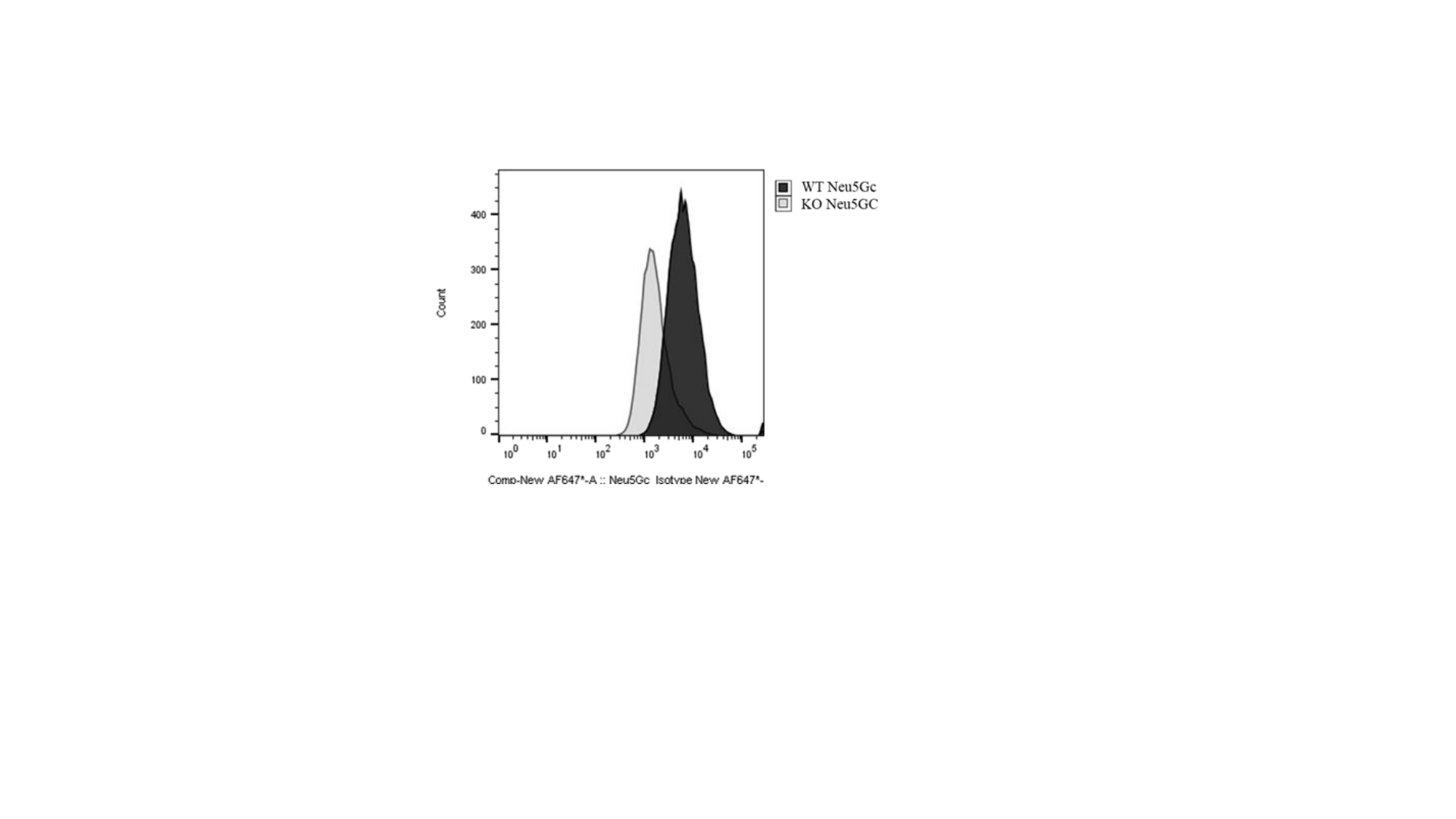

Supplement: Supplementary file 2 — Supplementary material 2 [file mmc2.pptx]
